# Effects of eHealth Interventions on Quality of Life and Psychological Outcomes in Cardiac Surgery Patients: Systematic Review and Meta-analysis

**DOI:** 10.2196/40090

**Published:** 2022-08-16

**Authors:** Ruping Ni, Maobai Liu, Shunmin Huang, Jing Yang

**Affiliations:** 1 Department of Pharmacy Fujian Medical University Union Hospital Fuzhou China; 2 College of Pharmacy Fujian Medical University Fuzhou China

**Keywords:** eHealth, eHealth intervention, cardiac surgery, depression, anxiety, quality of life, meta-analysis, heart disease, surgery, heart surgery, post-operative, postoperative, mental health, home care, digital health intervention, digital health, outcomes, psychological, physiological, physiology, psychology, compliance

## Abstract

**Background:**

Patients undergoing heart surgery may experience a range of physiological changes, and the postoperative recovery time is long. Patients and their families often have concerns about quality of life (QoL) after discharge. eHealth interventions may improve patient participation, ensure positive and effective health management, improve the quality of at-home care and the patient's quality of life, and reduce rates of depression.

**Objective:**

The purpose of this study was to evaluate the effects of eHealth interventions on the physiology, psychology, and compliance of adult patients after cardiac surgery to provide a theoretical basis for clinical practice.

**Methods:**

We conducted systematic searches of the following 4 electronic databases: PubMed, Embase, CINAHL, and the Cochrane Central Register of Controlled Trials. Mean (SD) values were used to calculate the pooled effect sizes for all consecutive data, including QoL, anxiety, and depression. Where the same results were obtained using different instruments, we chose the standardized mean difference with a 95% CI to represent the combined effect size; otherwise, the mean difference (MD) with a 95% CI was used. Odds ratios were used to calculate the combined effect size for all dichotomous data. The Cohen Q test for chi-square distribution and an inconsistency index (*I*^2^) were used to test for heterogeneity among the studies. We chose a fixed-effects model to estimate the effect size if there was no significant heterogeneity in the data (*I*^2^≤50%); otherwise, a random-effects model was used. The quality of the included studies was assessed using the Cochrane risk-of-bias tool for randomized trials (RoB 2).

**Results:**

The search identified 3632 papers, of which 19 met the inclusion criteria. In terms of physical outcomes, the score of the control group was lower than that of the intervention group (MD 0.15, 95% CI 0.03-0.27, *I*^2^=0%, *P*=.02). There was no significant difference in the mental outcomes between the intervention and control groups (MD 0.10, 95% CI –0.03 to 0.24, *I*^2^=46.4%, *P*=.14). The control group’s score was lower than that of the intervention group for the depression outcomes (MD –0.53, 95% CI –0.89 to –0.17, *I*^2^=57.1%, *P*=.004). Compliance outcomes improved in most intervention groups. The results of the sensitivity analysis were robust. Nearly half of the included studies (9/19, 47%) had a moderate to high risk of bias. The quality of the evidence was medium to low.

**Conclusions:**

eHealth improved the physical component of quality of life and depression after cardiac surgery; however, there was no statistical difference in the mental component of quality of life. The effectiveness of eHealth on patient compliance has been debated. Further high-quality studies on digital health are required.

**Trial Registration:**

PROSPERO CRD42022327305; https://www.crd.york.ac.uk/prospero/display_record.php?RecordID=327305

## Introduction

### Quality of Life and Cardiac Surgery

More than 1.5 million patients worldwide undergo heart surgery annually, and this number continues to grow [1]. Patients experience a series of psychophysiological changes before and after surgery. Preoperative anxiety and depression trigger the psychological response system, which in turn activates the endocrine and autonomic nervous systems, affecting postoperative outcomes, length of hospital stay, and quality of life [2,3]. Moreover, psychological changes related to chronic stress, such as anxiety and depression, can affect not only quality of life but also physiological parameters such as respiratory rate, heart rate, blood pressure, inflammatory markers, and brain activity, which may be detrimental to postoperative recovery [4-6]. The recovery period after cardiac surgery is relatively long, and most of the recovery processes, such as the healing of surgical wounds and the recovery of cardiac function, take place at home or in other facilities outside the hospital [7]. After cardiac surgery, patients and their families often have concerns regarding quality of life after discharge [8,9], since they will be solely responsible for at-home care [10,11]. Many problems can arise due to a lack of self-care knowledge and skills, and these problems increase with inadequate follow-up for patient education, counseling, and postoperative care [12].

### eHealth Interventions

In recent years, both health professionals and patients have been increasingly involved in eHealth [13], which includes mobile health, mobile and wireless technologies, health information technology, telemedicine, and personalized medicine, to improve clinical care, such as public health, health administration, and health-related education [14]. eHealth is often designed to support the achievement of health goals. With the increasing social demand for electronic technology, the use of mobile devices has the great potential to transform conventional health care and implement patient-centered initiatives [15-17]. Increased patient engagement is a key factor in eHealth and has the potential to motivate users and enable them to become more proactive and effective in managing their own health, ultimately improving quality of care [18]. The quality of health care has improved significantly with the use of telemedicine [19]. In addition, electronic medical interventions are already widely used in perioperative nursing [20].

Approximately 70% of patients consult the internet for information soon after learning about their upcoming surgery [21,22]. Studies [23,24] have reported that eHealth interventions for cardiac rehabilitation can also improve patients’ quality of life. These interventions provide continuous education regarding patient care and treatment, and offer counseling and support to at-home care providers while allowing access to vital information for patients, their families, and health care providers [25].

Many studies have evaluated the potential benefits of eHealth interventions in patients who have undergone cardiac surgery. However, to date, there has been no systematic evaluation of the effectiveness of these eHealth interventions compared to conventional care in terms of physiology, psychology, and compliance of adult patients after cardiac surgery. Therefore, we conducted this systematic review to assess the impact of eHealth interventions after cardiac surgery on quality of life, psychology, and compliance.

## Methods

### Design

This study was conducted and reported in accordance with the PRISMA (Preferred Reporting Items for Systematic Reviews and Meta-Analyses) statement (Multimedia Appendix 1) [26]. The systematic review protocol was registered in PROSPERO (International Prospective Register of Systematic Reviews; CRD42022327305).

### Search Strategy and Data Sources

The PubMed, Embase, CINAHL, and the Cochrane Central Register of Controlled Trials databases were searched from inception to April 2022. The search strategy consisted of 2 components: clinical situation (adult, cardiac surgery) and intervention type (health management using mobile phones, wearables, personal digital assistants, and other wireless devices). Relevant search items and combinations of Medical Subject Headings were used to identify trials related to eHealth and cardiac surgery. Searches were not limited to a specific geographic region, language, or period, but any literature without its full text was excluded. We exclusively included randomized controlled trials. The exact search terms used in each of the databases and the corresponding number of results are provided (Multimedia Appendix 2). EndNote 20 (Clarivate) was used for database management.

### Inclusion and Exclusion Criteria

The inclusion criteria were as follows: (1) patients aged 18 years or older at the time of heart surgery and studies that did not specify the type of heart surgery, (2) studies that evaluated any type of eHealth intervention, and (3) randomized controlled clinical studies.

Exclusion criteria were as follows: (1) studies where the full text could not be obtained, (2) insufficient clinical data that were reported in the form of meeting abstracts and did not provide detailed treatment methods or report the relevant results, and (3) duplicate studies.

### Document Screening and Data Extraction

Two researchers (RN and ML) independently performed the literature screening and data extraction according to the literature inclusion and exclusion criteria. Decisions on inclusion or exclusion were made by the researchers after a joint discussion of the results. Disagreements were resolved by a third party.

One researcher extracted the data using a literature data extraction table, and a second researcher confirmed the accuracy and authenticity of the data. The extracted content included study information (research topic, author, publication date, and region), baseline characteristics of the study participants (sample size and age), specific details of the intervention, follow-up time, and other outcome indicators (quality of life, anxiety and depression, cardiovascular events, treatment, and medication compliance).

### Data Analyses

Mean (SD) values were used to calculate the pooled effect sizes for all consecutive data, including quality of life and depression. When measuring the same outcome using different instruments, we chose the standardized mean difference with a 95% CI to represent the combined effect size; otherwise, we used the mean difference (MD) with a 95% CI to represent the combined effect size. Odds ratios were used to calculate the combined effect size for all dichotomous data. The Cohen Q test for chi-square distribution and an inconsistency index (*I*^2^) were used to test for heterogeneity among the studies. We selected a fixed-effects model to estimate the effect size if there was no significant heterogeneity in the data (*I*^2^≤50%). Otherwise, a random effects model was used. A sensitivity analysis was performed using the leave-one-out method. All meta-analyses were performed using the Stata software (version 15.1; StataCorp).

### Quality Assessment

Before analyzing the extracted data, 2 researchers independently assessed the quality of the included studies. A discussion with a third reviewer was conducted until a consensus was reached and disagreements were resolved. The quality of each study was assessed according to the guidelines provided by the Cochrane risk-of-bias tool for randomized trials, version 2.0 (RoB 2) [27]. The overall quality of evidence for each outcome was assessed using the GRADE (Grading of Recommendations, Assessment, Development and Evaluations) approach [28].

## Results

### Identification of Studies

**Figure 1:**
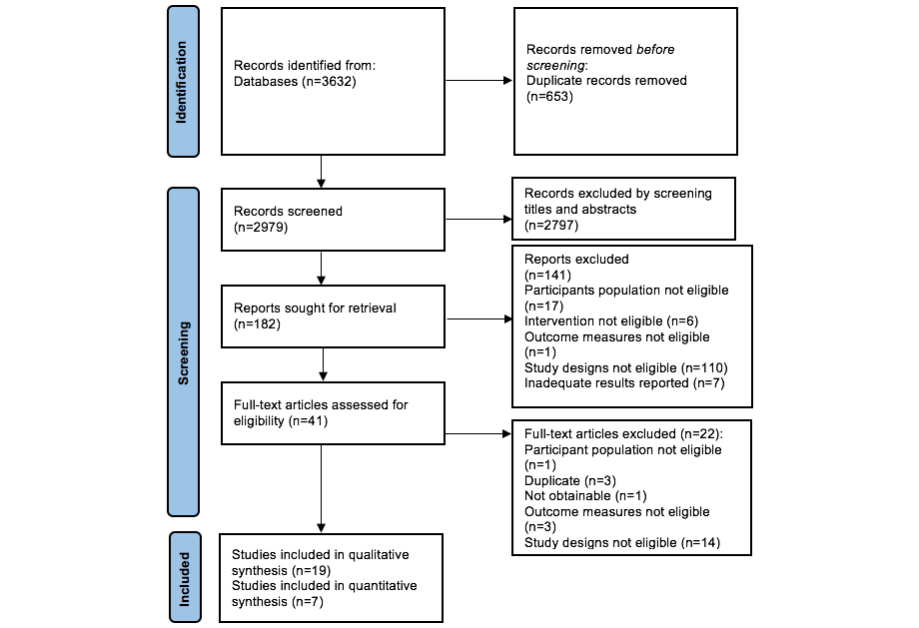
The PRISMA (Preferred Reporting Items for Systematic Reviews and Meta-Analyses) flowchart.

The PRISMA flowchart in Figure 1 summarizes the search results and selection process for all studies included in our synthesis. A total of 3632 articles were retrieved through a systematic literature search. After removing duplicate studies, the remaining 2979 records were screened. After reading 41 eligible full-text articles, 22 were excluded, and 19 were selected [29-47]. A summary of the study characteristics and participant demographics are presented in Multimedia Appendix 3 [29-47].

### Meta-analysis

#### Primary Outcome

##### Quality of Life

**Figure 2:**
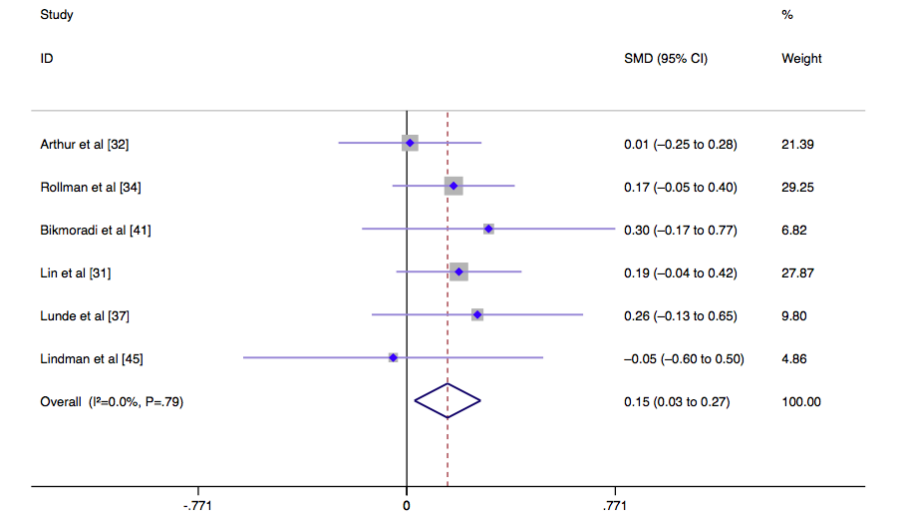
Forest plot of the effect of physical outcomes of quality of life after cardiac surgery. SMD: standardized mean difference.

**Figure 3:**
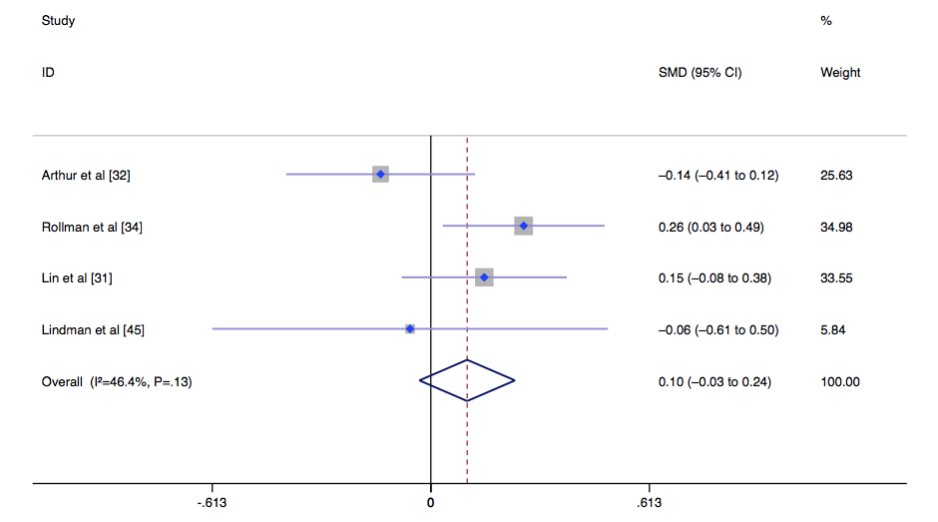
Forest plot of the effect of mental outcomes of quality of life after cardiac surgery. SMD: standardized mean difference.

The fixed-effects analysis model was used to analyze the physical and mental outcomes of quality of life. In terms of physical outcomes, the scores of the control group were lower than those of the intervention group (MD 0.15, 95% CI 0.03-0.27, *I*^2^=0%, *P*=.02) (Figure 2). However, there was no significant difference in the mental outcomes between the intervention and control groups (MD 0.10, 95% CI –0.03 to 0.24, *I*^2^=46.4%, *P*=.14) (Figure 3).

##### Depression

**Figure 4:**
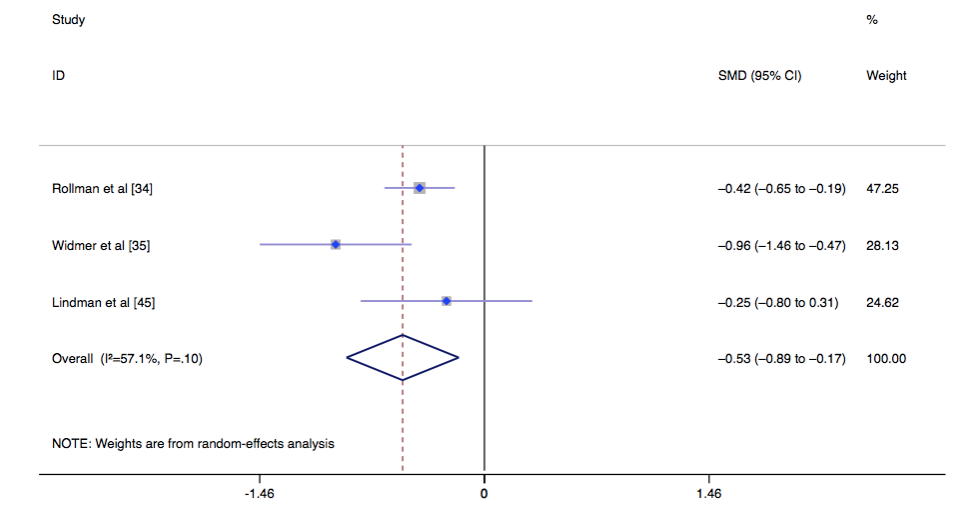
Forest plot of the effect of depression after cardiac surgery. SMD: standardized mean difference.

To evaluate depression outcomes, we used a random-effects analysis model. The score of the control group was lower than that of the intervention group (MD –0.53, 95% CI –0.89 to –0.17, *I*^2^=57.1%, *P*=.004) (Figure 4). Owing to sparse data, there was no subgroup analysis of the main outcome indicators based on a follow-up period of 3 months.

#### Other Outcomes

Three studies [29,30,42] reported no significant difference in the improvement of anxiety between the intervention and control groups. The occurrence of mortality was reported in 4 studies [31,32,36,39], of which 1 study [31] showed a statistically significant reduction in mortality in the intervention group, while the other 3 studies reported different conclusions. A total of 4 studies [30,35,39,42] reported no statistical significance for readmission between the intervention and control groups. Among the 3 studies [31,39,43] that reported on compliance, 2 studies [31,43] showed better compliance in the experimental group compared to the control group. However, 1 study [39] showed no statistical difference in compliance between the two groups. Two studies [35,37] indicated that none of the 4 lipid indexes had statistical significance. We generated 2 forest plots to show the effects of eHealth on other outcomes (Multimedia Appendices 4 and 5). Most of the studies had no significant differences in their results, apart from those for compliance, bleeding events, secondary prophylactic medication, patient satisfaction, and time in the therapeutic range (Multimedia Appendices 4 and 5).

### Sensitivity Analysis

A sensitivity analysis of quality of life and depressive outcomes was performed using the leave-one-out method, as shown in Multimedia Appendix 6 [31,32,34,35,37,41,45], and the results were consistent.

### Quality Assessment

**Figure 5:**
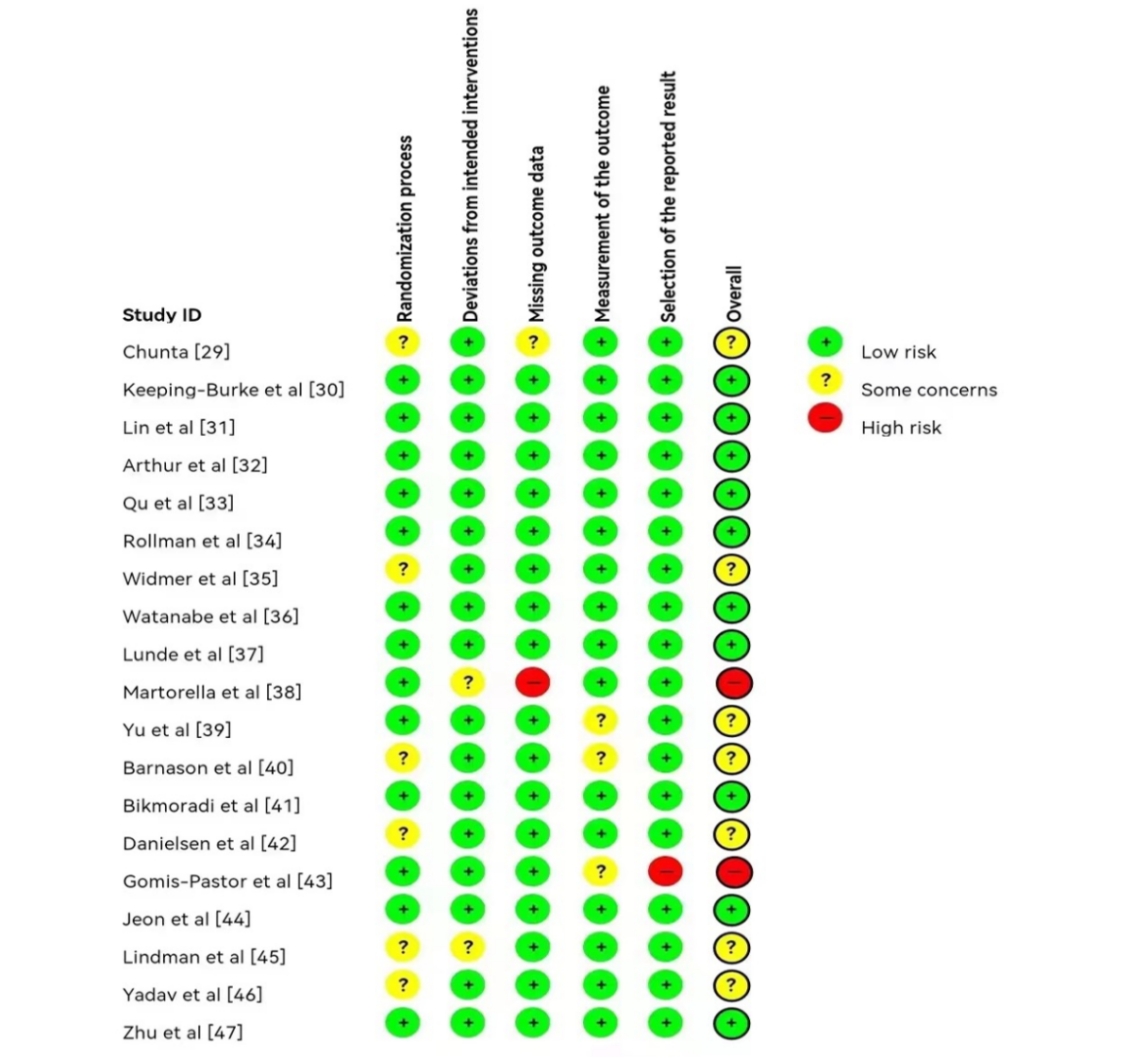
A risk-of-bias map using the Cochrane systematic evaluation method to assess the quality of the included randomized controlled trials.

RoB 2 [27] was used for quality evaluation. Overall, the included studies had a low to moderate risk of bias, as shown in Figure 5. Most articles did not clearly report the randomization process. The overall quality of evidence for each outcome was assessed using the GRADE approach [28]. The quality rates of each outcome are shown in Multimedia Appendix 7. In summary, although the quality of some outcomes was moderate, the overall quality was low.

## Discussion

### Principal Findings

In this systematic review, we assessed the impact of eHealth interventions on cardiac surgery recovery based on the results of 19 studies. These studies reported at least an equal (n=6) [29,30,33,38,39,42] or positive effect (n=13) [31,32,34-37,40,41,43-47] of the eHealth intervention compared to conventional care. According to the results of the meta-analysis, when compared with the control group, the eHealth intervention group showed an improvement in both the physical component of quality of life and the depressive status of patients after cardiac surgery. The mental component of quality of life was not significantly different in the two groups. This may be related to the shorter follow-up period of the included studies. Lin et al [31] showed that an effect on quality of life was not observed until the follow-up after 18 months, which was not long enough for most of the studies we included.

van der Meij et al [48] showed that eHealth interventions have similar effects on different types of postoperative outcome measurements. The results for physical and psychological indicators were comparable. Therefore, we only conducted a meta-analysis on quality of life and depression. Study results on patients with different types of heart surgery, eHealth interventions, and measured outcomes varied widely. Due to the lack of reported data and heterogeneity, analysis of the other results using statistical methods was not performed. The economics of eHealth interventions have also not been studied yet. In terms of medication adherence, 2 of the 3 studies reported improved medication adherence after eHealth intervention [31,43]. Fewer than 10 studies were included in the quantitative analysis for each outcome; therefore, publication bias analysis was not performed. However, the findings should be interpreted with caution, as the overall quality of the body of evidence was low to moderate because of the risk of bias in the included studies.

Patients undergoing different cardiac surgeries have different postoperative needs. The studies in this review included eHealth interventions for medication education, consultation, follow-up, postoperative exercise, and rehabilitation. eHealth interventions were also used specifically for postoperative pain [38], anticoagulant management [44,47], and secondary drug prevention [33,35,39]. Martorella et al [38] revealed that patients in the experimental group did not experience less intense pain but reported significantly less pain interference when breathing or coughing (*P*=.04). However, the experimental group consumed more opioid medication (mean 31.2, SD 23.2 mg) than the control group (mean 18.8, SD 15.3 mg; *P*=.001). Two studies [44,47] showed that the use of eHealth improved efficacy in maintaining the therapeutic range of prothrombin time. Another study [44] showed improvement in self-management knowledge, self-efficacy, and improved behavior of patients undergoing cardiac valve replacement, as well as reduced adverse events for bleeding thrombosis [47] through eHealth intervention. Qu et al [33] showed that eHealth interventions have limited ability to increase prescription rates for statins or other drugs. Widmer et al [35] showed that eHealth interventions can improve the secondary prevention of cardiovascular diseases. Yu et al [39] showed that the intervention group had no significant impact on mortality, major adverse cerebrovascular events, and cardiovascular rehospitalization, which may be related to low patient participation.

### Limitations

First, due to sparse data, there was no subgroup analysis of the main outcomes according to follow-up time, nor was there a comparative analysis of the pros and cons of different types of electronic interventions and different types of cardiac surgery on postoperative effects. Moreover, due to the limited number of included studies and the lack of publication bias analysis, the number of measured depression outcomes was small, and there was a possibility of deviation. Finally, allocation hiding was not explicitly reported in most of the included studies. The quality of the study outcomes was relatively low, and more high-quality randomized controlled trials should be included in the future.

### Comparison With Prior Work

According to our literature review, there have been many studies on the clinical application of eHealth interventions. However, to our knowledge, there is no systematic study on the impact of electronic interventions on patients after cardiac surgery. This is the first published systematic evaluation of the effects of using eHealth on patients who have undergone cardiac surgery. We ensured the use of robust methodology to conduct this review by following the PRISMA guidelines [26].

Among the published systematic evaluations, studies on the application of electronic interventions included patients with cancer, respiratory diseases, and arthritis. In terms of quality of life, 3 studies [49-51] reported that electronic interventions were ineffective, but 7 [52-58] reported improvement in quality of life. Two articles [51,57] reported that electronic intervention was ineffective in relieving anxiety, and another [59] showed mixed views. Electronic intervention was reported to be ineffective for depression in 2 studies [51,60], whereas 3 articles [54,55,59] provided mixed conclusions. There were mixed results regarding the effect of pain relief, with some studies [50,51] indicating no effect on pain relief, and others [55,61] reporting the opposite. Seven studies reported on patient compliance: 2 studies [51,62] showed no statistical significance in improving patient compliance, 3 studies [63-65] showed a positive impact, and according to the remaining 2 studies [66,67], the impact was uncertain. Another study [68] indicated that electronic intervention could effectively improve maximum aerobic capacity and alter cardiovascular risk factors. In addition, we found that the effectiveness of electronic interventions may be related to the disease type. eHealth interventions showed positive effects on the outcome of some patients [52-55], but studies reporting on patients with cancer [49] and patients with arthritis [50,51] reported negative results.

There are many different types of cardiac surgery, such as valve replacement, bypass, and heart transplantation. The various results relating to different disease types might indicate that the effect of eHealth intervention may vary according to patient type. More high-quality studies are needed to verify these findings.

### Conclusions

Based on this systematic review, the eHealth intervention group showed improvement in both the physical component of quality of life and depressive status after cardiac surgery, but the positive effects of the intervention were small. Moreover, the mental component of quality of life was not significantly different between the two groups. The overall quality of the evidence was low to medium. The compliance outcomes improved in most intervention groups. In the future, higher-quality randomized controlled studies of eHealth interventions are needed to provide more evidence for clinical practice.

## Appendix

Multimedia Appendix 1 The PRISMA (Preferred Reporting Items for Systematic Reviews and Meta-Analyses) checklist.

Multimedia Appendix 2 Search terms.

Multimedia Appendix 3 Baseline characteristics of the 19 studies selected for the meta-analysis.

Multimedia Appendix 4 Forest plot of the effect of eHealth on other cardiac postoperative outcomes (continuous data).

Multimedia Appendix 5 Forest plot of the effect of eHealth on other cardiac postoperative outcomes (dichotomous data).

Multimedia Appendix 6 Influence analysis.

Multimedia Appendix 7 The overall quality of the evidence for each outcome.

## Abbreviations

GRADE: Grading of Recommendations, Assessment, Development and Evaluations
MD: mean difference
PRISMA: Preferred Reporting Items for Systematic Reviews and Meta-Analyses
PROSPERO: International Prospective Register of Systematic Reviews
QoL: quality of life
