# Supplementary material for: Effects of eHealth Interventions on Quality of Life and Psychological Outcomes in Cardiac Surgery Patients: Systematic Review and Meta-analysis
Source: J Med Internet Res. 2022 Aug 16;24(8):e40090. doi: 10.2196/40090 (PMC9428777; doi:10.2196/40090)
Supplement: Multimedia Appendix 2 [file jmir_v24i8e40090_app2.docx]

Multimedia Appendix 2

(Effects of e-health interventions on quality of life and psychological outcomes in cardiac surgery patients: a systematic review and meta-analysis)

##### Textbox S1: Search terms

| The following terms were used to search the articles in PubMed. It should be noted that we initially screened all diabetes-related complications and decided to focus on gestational diabetes finally.  **1,PUBMED**  ((((((((((((((((((((((((((((((((((((((((((((((Surgery, Cardiac[Title/Abstract]) OR (Surgery, Heart[Title/Abstract])) OR (Heart Surgery[Title/Abstract])) OR (Cardiac Surgery[Title/Abstract])) OR (Procedure, Cardiac Surgical[Title/Abstract])) OR (Procedures, Cardiac Surgical[Title/Abstract])) OR (Surgical Procedure, Cardiac[Title/Abstract])) OR (Surgical Procedures, Cardiac[Title/Abstract])) OR (Surgical Procedures, Heart[Title/Abstract])) OR (Cardiac Surgical Procedure[Title/Abstract])) OR (Heart Surgical Procedures[Title/Abstract])) OR (Procedure, Heart Surgical[Title/Abstract])) OR (Procedures, Heart Surgical[Title/Abstract])) OR (Surgical Procedure, Heart[Title/Abstract])) OR (Heart Surgical Procedure[Title/Abstract])) OR (Arterial Switch Operation[Title/Abstract])) OR (Cardiac Valve Annuloplasty[Title/Abstract])) OR (Mitral Valve Annuloplasty[Title/Abstract])) OR (Cardiomyoplasty[Title/Abstract])) OR (Heart Arrest, Induced[Title/Abstract])) OR (Circulatory Arrest, Deep Hypothermia Induced[Title/Abstract])) OR (Heart Bypass, Right[Title/Abstract])) OR (Fontan Procedure[Title/Abstract])) OR (Heart Massage[Title/Abstract])) OR (Heart Transplantation[Title/Abstract])) OR (Heart-Lung Transplantation[Title/Abstract])) OR (Heart Valve Prosthesis Implantation[Title/Abstract])) OR (Transcatheter Aortic Valve Replacement[Title/Abstract])) OR (Maze Procedure[Title/Abstract])) OR (Myocardial Revascularization[Title/Abstract])) OR (Angioplasty, Balloon, Coronary[Title/Abstract])) OR (Atherectomy, Coronary[Title/Abstract])) OR (Coronary Artery Bypass +[Title/Abstract])) OR (Transmyocardial Laser Revascularization[Title/Abstract])) OR (Norwood Procedures[Title/Abstract])) OR (Fontan Procedure[Title/Abstract])) OR (Pericardial Window Techniques[Title/Abstract])) OR (Pericardiectomy[Title/Abstract])) OR (Pericardiocentesis[Title/Abstract])) OR (cardiac surgery[Title/Abstract])) OR (heart operation[Title/Abstract])) OR (cardiosurgery[Title/Abstract])) OR (Heart surgery[Title/Abstract])) OR (open-heart surgery[Title/Abstract])) OR (open heart surgery[Title/Abstract])) OR ("Cardiac Surgical Procedures"[Mesh]) ) AND ((((((((((((((((telemedicine[MeSH Terms]) OR (Mobile Health[Title/Abstract])) OR (Health, Mobile[Title/Abstract])) OR (mHealth[Title/Abstract])) OR (Telehealth[Title/Abstract])) OR (eHealth[Title/Abstract])) OR (((((Smartphone[MeSH Terms]) OR (Smartphones[Title/Abstract])) OR (Smart Phones[Title/Abstract])) OR (Smart Phone[Title/Abstract])) OR (Phones, Smart[Title/Abstract]))) OR ((((((((((((((((((((((((((((Mobile Applications[MeSH Terms]) OR (Application, Mobile[Title/Abstract])) OR (Applications, Mobile[Title/Abstract])) OR (Mobile Application[Title/Abstract])) OR (Mobile Apps[Title/Abstract])) OR (App, Mobile[Title/Abstract])) OR (Apps, Mobile[Title/Abstract])) OR (Mobile App[Title/Abstract])) OR (Portable Software Apps[Title/Abstract])) OR (App, Portable Software[Title/Abstract])) OR (Portable Software App[Title/Abstract])) OR (Software App, Portable[Title/Abstract])) OR (Portable Software Applications[Title/Abstract])) OR (Application, Portable Software[Title/Abstract])) OR (Portable Software Application[Title/Abstract])) OR (Software Application, Portable[Title/Abstract])) OR (Smartphone Apps[Title/Abstract])) OR (App, Smartphone[Title/Abstract])) OR (Apps, Smartphone[Title/Abstract])) OR (Smartphone App[Title/Abstract])) OR (Portable Electronic Apps[Title/Abstract])) OR (App, Portable Electronic[Title/Abstract])) OR (Electronic App, Portable[Title/Abstract])) OR (Portable Electronic App[Title/Abstract])) OR (Portable Electronic Applications[Title/Abstract])) OR (Application, Portable Electronic[Title/Abstract])) OR (Electronic Application, Portable[Title/Abstract])) OR (Portable Electronic Application[Title/Abstract]))) OR (((((((((((((((((((((((((((((((((Cell Phone[MeSH Terms]) OR (Phone, Cell[Title/Abstract])) OR (Phones, Cell[Title/Abstract])) OR (Cellular Phone[Title/Abstract])) OR (Cellular Phones[Title/Abstract])) OR (Phone, Cellular[Title/Abstract])) OR (Phones, Cellular[Title/Abstract])) OR (Telephone, Cellular[Title/Abstract])) OR (Cellular Telephone[Title/Abstract])) OR (Cellular Telephones[Title/Abstract])) OR (Telephones, Cellular[Title/Abstract])) OR (Cell Phones[Title/Abstract])) OR (Portable Cellular Phone[Title/Abstract])) OR (Cellular Phone, Portable[Title/Abstract])) OR (Cellular Phones, Portable[Title/Abstract])) OR (Portable Cellular Phones[Title/Abstract])) OR (Transportable Cellular Phone[Title/Abstract])) OR (Cellular Phone, Transportable[Title/Abstract])) OR (Cellular Phones, Transportable[Title/Abstract])) OR (Transportable Cellular Phones[Title/Abstract])) OR (Mobile Phone[Title/Abstract])) OR (Mobile Phones[Title/Abstract])) OR (Phone, Mobile[Title/Abstract])) OR (Phones, Mobile[Title/Abstract])) OR (Mobile Telephone[Title/Abstract])) OR (Mobile Telephones[Title/Abstract])) OR (Telephone, Mobile[Title/Abstract])) OR (Telephones, Mobile[Title/Abstract])) OR (Car Phone[Title/Abstract])) OR (Car Phones[Title/Abstract])) OR (Phone, Car[Title/Abstract])) OR (Phones, Car[Title/Abstract])) OR (Telephone[Title/Abstract]))) OR (((((((Electronic Mail[MeSH Terms]) OR (Mail, Electronic[Title/Abstract])) OR (Email[Title/Abstract])) OR (Emails[Title/Abstract])) OR (E-Mail[Title/Abstract])) OR (E Mail[Title/Abstract])) OR (E-Mails[Title/Abstract]))) OR (((((((((((((((((((Internet-Based Intervention[MeSH Terms]) OR (Internet Based Intervention[Title/Abstract])) OR (Internet-Based Interventions[Title/Abstract])) OR (Intervention, Internet-Based[Title/Abstract])) OR (Interventions, Internet-Based[Title/Abstract])) OR (Web-based Intervention[Title/Abstract])) OR (Intervention, Web-based[Title/Abstract])) OR (Interventions, Web-based[Title/Abstract])) OR (Web based Intervention[Title/Abstract])) OR (Web-based Interventions[Title/Abstract])) OR (Online Intervention[Title/Abstract])) OR (Intervention, Online[Title/Abstract])) OR (Interventions, Online[Title/Abstract])) OR (Online Interventions[Title/Abstract])) OR (Internet Intervention[Title/Abstract])) OR (Internet Interventions[Title/Abstract])) OR (Intervention, Internet[Title/Abstract])) OR (Interventions, Internet[Title/Abstract])) OR (Internet[Title/Abstract]))) OR ((((((((((Text Messaging[MeSH Terms]) OR (SMS[Title/Abstract])) OR (Messaging, Text[Title/Abstract])) OR (Texting[Title/Abstract])) OR (Textings[Title/Abstract])) OR (Short Message Service[Title/Abstract])) OR (Text Messages[Title/Abstract])) OR (Message, Text[Title/Abstract])) OR (Messages, Text[Title/Abstract])) OR (Text Message[Title/Abstract]))) OR (((((((Distance Counseling[MeSH Terms]) OR (Counseling, Distance[Title/Abstract])) OR (E-Therapy[Title/Abstract])) OR (E Therapy[Title/Abstract])) OR (E-Therapies[Title/Abstract])) OR (E-Counseling[Title/Abstract])) OR (E Counseling[Title/Abstract]))) OR ((((((((((((telecommunications system[Title/Abstract]) OR (telecommunication system[Title/Abstract])) OR (web[Title/Abstract])) OR (web communication[Title/Abstract])) OR (wireless[Title/Abstract])) OR (cloud-based[Title/Abstract])) OR (web-based[Title/Abstract])) OR (APP[Title/Abstract])) OR (short-messages-based interaction[Title/Abstract])) OR (mobile phone text messaging[Title/Abstract])) OR (phone call[Title/Abstract])) OR (internet-based[Title/Abstract]))) OR ((((((((((((((distance[Title/Abstract]) AND (counseling[Title/Abstract]) ) OR (telecommunications system[Title/Abstract])) OR (telecommunication system[Title/Abstract])) OR (web[Title/Abstract])) OR (web communication[Title/Abstract])) OR (wireless[Title/Abstract])) OR (cloud-based[Title/Abstract])) OR (web-based[Title/Abstract])) OR (APP[Title/Abstract])) OR (short-messages-based interaction[Title/Abstract])) OR (mobile phone text messaging[Title/Abstract])) OR (phone call[Title/Abstract])) OR (internet-based[Title/Abstract])))  **2,Embase**  Session Results  .......................................................  No. Query Results Results Date  #26. #5 AND #25 895 11 Apr 2022  #25. #6 OR #9 OR #12 OR #15 OR #18 OR #21 OR #24 138,218 11 Apr 2022  #24. #22 OR #23 480 11 Apr 2022  #23. 'distance counseling':ab,ti OR 'online 181 11 Apr 2022  counseling':ab,ti OR 'remote counseling':ab,ti OR  'tele counseling':ab,ti  #22. 'e-counseling'/exp 364 11 Apr 2022  #21. #19 OR #20 6,935 11 Apr 2022  #20. texting:ab,ti 1,476 11 Apr 2022  #19. 'text messaging'/exp 6,558 11 Apr 2022  #18. #16 OR #17 2,986 11 Apr 2022  #17. 'internet-based intervention':ab,ti OR 'internet 1,604 11 Apr 2022  intervention':ab,ti OR 'online-based  intervention':ab,ti OR 'online  intervention':ab,ti OR 'web intervention':ab,ti  #16. 'web-based intervention'/exp 1,645 11 Apr 2022  #15. #13 OR #14 40,150 11 Apr 2022  #14. 'electronic mail':ab,ti OR 'electronic 32,920 11 Apr 2022  messaging':ab,ti OR email:ab,ti OR 'mail,  electronic':ab,ti OR 'mailing, electronic':ab,ti  #13. 'e-mail'/exp 26,476 11 Apr 2022  #12. #10 OR #11 20,588 11 Apr 2022  #11. 'mobile app':ab,ti OR 'mobile applications':ab,ti 5,827 11 Apr 2022  OR 'mobile apps':ab,ti OR 'portable software  app':ab,ti OR 'portable software  application':ab,ti OR 'portable software  applications':ab,ti OR 'portable software  apps':ab,ti OR 'tablet application':ab,ti  #10. 'mobile application'/exp 19,528 11 Apr 2022  #9. #7 OR #8 23,506 11 Apr 2022  #8. 'smartphone'/exp 19,990 11 Apr 2022  #7. 'smart phone':ab,ti OR smartphones:ab,ti 8,951 11 Apr 2022  #6. #3 OR #4 58,901 11 Apr 2022  #5. #1 OR #2 442,970 11 Apr 2022  #4. 'tele medicine':ab,ti OR 'virtual medicine':ab,ti 362 11 Apr 2022  #3. 'telemedicine'/exp 58,825 11 Apr 2022  #2. 'cardiac surgery':ab,ti OR 'cardiac surgical 68,162 11 Apr 2022  procedures':ab,ti OR cardiosurgery:ab,ti OR  'heart operation':ab,ti OR 'myocardial  resection':ab,ti OR 'surgery, heart':ab,ti  #1. 'heart surgery'/exp 430,732 11 Apr 2022  .......................................................  **3,Cochrane Central Register of Controlled Trials**  Date Run: 11/04/2022 18:47:12  ID Search Hits  #1 MeSH descriptor: [Thoracic Surgery] explode all trees 174  #2 (Surgery, Cardiac):ti,ab,kw OR (Cardiac; Heart Surgery):ti,ab,kw OR (Cardiac Surgery):ti,ab,kw OR (Surgery, Heart):ti,ab,kw AND (Surgery, Thoracic):ti,ab,kw 20063  #3 #1 or #2 20172  #4 MeSH descriptor: [Telemedicine] explode all trees 3174  #5 (eHealth):ti,ab,kw OR (Telehealth):ti,ab,kw OR (Mobile Health):ti,ab,kw OR (mHealth):ti,ab,kw OR (Health, Mobile):ti,ab,kw 9886  #6 MeSH descriptor: [Smartphone] explode all trees 561  #7 (Smart Phone):ti,ab,kw OR (Smart Phones):ti,ab,kw OR (Smartphones):ti,ab,kw OR (Phones, Smart):ti,ab,kw 1901  #8 MeSH descriptor: [Mobile Applications] explode all trees 1020  #9 (App*, Smartphone*):ti,ab,kw OR (App, Portable Electronic):ti,ab,kw OR (Portable Electronic Application*):ti,ab,kw OR (Portable Electronic App*):ti,ab,kw OR (Electronic Application, Portable):ti,ab,kw 132  #10 (Electronic App, Portable):ti,ab,kw OR (Application, Portable Electronic):ti,ab,kw OR (Portable Software Applications):ti,ab,kw OR (Portable Software Application):ti,ab,kw OR (Software App):ti,ab,kw 487  #11 (Portable; App):ti,ab,kw OR (Portable Software):ti,ab,kw OR (Application, Portable Software):ti,ab,kw OR (Portable Software App*):ti,ab,kw OR (Software Application, Portable):ti,ab,kw 246  #12 (App*, Mobile):ti,ab,kw OR (Mobile App*):ti,ab,kw OR (Mobile Application):ti,ab,kw OR (Application*, Mobile):ti,ab,kw 7698  #13 MeSH descriptor: [Electronic Mail] explode all trees 358  #14 (E-Mail*):ti,ab,kw OR (E Mail):ti,ab,kw OR (Email*):ti,ab,kw OR (Mail, Electronic):ti,ab,kw 6236  #15 MeSH descriptor: [Internet-Based Intervention] explode all trees 322  #16 (Intervention*, Online):ti,ab,kw OR (Web-based Intervention*):ti,ab,kw OR (Online Intervention*):ti,ab,kw OR (Intervention*, Internet-Based):ti,ab,kw OR (Web based Intervention*):ti,ab,kw 16235  #17 (Intervention*, Internet):ti,ab,kw OR (Intervention*, Internet-Based):ti,ab,kw OR (Internet Intervention*):ti,ab,kw OR (Intervention*, Web-based):ti,ab,kw OR (Internet-Based Intervention*):ti,ab,kw 8661  #18 MeSH descriptor: [Text Messaging] explode all trees 1101  #19 (Short Message Service):ti,ab,kw OR (Message*, Text):ti,ab,kw OR (Text Message*):ti,ab,kw OR (Textings):ti,ab,kw OR (Messaging, Text):ti,ab,kw 5340  #20 MeSH descriptor: [Distance Counseling] explode all trees 23  #21 (E Therapy):ti,ab,kw OR (Counseling, Distance):ti,ab,kw OR (E-Therapie*):ti,ab,kw OR (E-Counseling):ti,ab,kw OR (E Counseling):ti,ab,kw 58586  #22 #4 or #5 or #6 or #7 or #8 or #9 or #10 or #11 or #12 or #13 or #14 or #15 or #16 or #17 or #18 or #19 or #20 or #21 93757  #23 #3 and #22 830  **4, CINAHL**  Monday, April 11, 2022 2:35:55 PM  S1  AB ( (((((((((((((telemedicine) OR (Mobile Health)) OR (Health, Mobile)) OR (mHealth)) OR (Telehealth)) OR (eHealth)) OR (((((Smartphone) OR (Smartphones)) OR (Smart Phones)) OR (Smart Phone)) OR (Phones, Smart))) OR ((((((((((((((((((((((((((((Mobile Applications) OR (Application, Mobile)) OR (Applications, Mobile)) OR (Mobile Application)) OR (Mobile Apps)) OR (App, Mobile)) OR (Apps, Mobile)) OR (Mobile App)) OR (Portable Software Apps)) OR (App, Portable Software)) OR (Portable Software App)) OR (Software App, Portable)) OR (Portable Software Applications)) OR (Application, Portable Software)) OR (Portable Software Application)) OR (Software Application, Portable)) OR (Smartphone Apps)) OR (App, Smartphone)) OR (Apps, Smartphone)) OR (Smartphone App)) OR (Portable Electronic Apps)) OR (App, Portable Electronic)) OR (Electronic App, Portable)) OR (Portable Electronic App)) OR (Portable Electronic Applications)) OR (Application, Portable Electronic)) OR (Electronic Application, Portable)) OR (Portable Electronic Application))) OR (((((((((((((((((((((((((((((((((Cell Phone) OR (Phone, Cell)) OR (Phones, Cell)) OR (Cellular Phone)) OR (Cellular Phones)) OR (Phone, Cellular)) OR (Phones, Cellular)) OR (Telephone, Cellular)) OR (Cellular Telephone)) OR (Cellular Telephones)) OR (Telephones, Cellular)) OR (Cell Phones)) OR (Portable Cellular Phone)) OR (Cellular Phone, Portable)) OR (Cellular Phones, Portable)) OR (Portable Cellular Phones)) OR (Transportable Cellular Phone)) OR (Cellular Phone, Transportable)) OR (Cellular Phones, Transportable)) OR (Transportable Cellular Phones)) OR (Mobile Phone)) OR (Mobile Phones)) OR (Phone, Mobile)) OR (Phones, Mobile)) OR (Mobile Telephone)) OR (Mobile Telephones)) OR (Telephone, Mobile)) OR (Telephones, Mobile)) OR (Car Phone)) OR (Car Phones)) OR (Phone, Car)) OR (Phones, Car)) OR (Telephone))) OR (((((((Electronic Mail) OR (Mail, Electronic)) OR (Email)) OR (Emails)) OR (E-Mail)) OR (E Mail)) OR (E-Mails))) OR (((((((((((((((((((Internet-Based Intervention) OR (Internet Based Intervention)) OR (Internet-Based Interventions)) OR (Intervention, Internet-Based)) OR (Interventions, Internet-Based)) OR (Web-based Intervention)) OR (Intervention, Web-based)) OR (Interventions, Web-based)) OR (Web based Intervention)) OR (Web-based Interventions)) OR (Online Intervention)) OR (Intervention, Online)) OR (Interventions, Online)) OR (Online Interventions)) OR (Internet Intervention)) OR (Internet Interventions)) OR (Intervention, Internet)) OR (Interventions, Internet)) OR (Internet))) OR ((((((((((Text Messaging) OR (SMS)) OR (Messaging, Text)) OR (Texting)) OR (Textings)) OR (Short Message Service)) OR (Text Messages)) OR (Message, Text)) OR (Messages, Text)) OR (Text Message))) OR (((((((Distance Counseling) OR (Counseling, Distance)) OR (E-Therapy)) OR (E Therapy)) OR (E-Therapies)) OR (E-Counseling)) OR (E Counseling))) OR (((((((((((((telecommunications system) OR (telecommunication system)) OR (web)) OR (web communication)) OR (wireless)) OR (cloud-based)) OR (web-based)) OR (APP)) OR (short-messages-based interaction)) OR (mobile phone text messaging)) OR (phone call)) OR (internet-based)) OR ((distance) AND (counseling))) ) AND AB ( (((((((((((((((((((((((((((((((((((((((((((((Cardiac Surgical Procedures) OR (Surgery, Cardiac)) OR (Surgery, Heart)) OR (Heart Surgery)) OR (Cardiac Surgery)) OR (Procedure, Cardiac Surgical)) OR (Procedures, Cardiac Surgical)) OR (Surgical Procedure, Cardiac)) OR (Surgical Procedures, Cardiac)) OR (Surgical Procedures, Heart)) OR (Cardiac Surgical Procedure)) OR (Heart Surgical Procedures)) OR (Procedure, Heart Surgical)) OR (Procedures, Heart Surgical)) OR (Surgical Procedure, Heart)) OR (Heart Surgical Procedure)) OR (Arterial Switch Operation)) OR (Cardiac Valve Annuloplasty)) OR (Mitral Valve Annuloplasty)) OR (Cardiomyoplasty)) OR (Heart Arrest, Induced)) OR (Circulatory Arrest, Deep Hypothermia Induced)) OR (Heart Bypass, Right)) OR (Fontan Procedure)) OR (Heart Massage)) OR (Heart Transplantation)) OR (Heart-Lung Transplantation)) OR (Heart Valve Prosthesis Implantation)) OR (Transcatheter Aortic Valve Replacement)) OR (Maze Procedure)) OR (Myocardial Revascularization)) OR (Angioplasty, Balloon, Coronary)) OR (Atherectomy, Coronary)) OR (Coronary Artery Bypass +)) OR (Transmyocardial Laser Revascularization)) OR (Norwood Procedures)) OR (Fontan Procedure)) OR (Pericardial Window Techniques)) OR (Pericardiectomy)) OR (Pericardiocentesis)) OR (cardiac surgery)) OR (heart operation)) OR (cardiosurgery)) OR (Heart surgery)) OR (open-heart surgery)) OR (open heart surgery) )  S2  TI ( (((((((((((((telemedicine) OR (Mobile Health)) OR (Health, Mobile)) OR (mHealth)) OR (Telehealth)) OR (eHealth)) OR (((((Smartphone) OR (Smartphones)) OR (Smart Phones)) OR (Smart Phone)) OR (Phones, Smart))) OR ((((((((((((((((((((((((((((Mobile Applications) OR (Application, Mobile)) OR (Applications, Mobile)) OR (Mobile Application)) OR (Mobile Apps)) OR (App, Mobile)) OR (Apps, Mobile)) OR (Mobile App)) OR (Portable Software Apps)) OR (App, Portable Software)) OR (Portable Software App)) OR (Software App, Portable)) OR (Portable Software Applications)) OR (Application, Portable Software)) OR (Portable Software Application)) OR (Software Application, Portable)) OR (Smartphone Apps)) OR (App, Smartphone)) OR (Apps, Smartphone)) OR (Smartphone App)) OR (Portable Electronic Apps)) OR (App, Portable Electronic)) OR (Electronic App, Portable)) OR (Portable Electronic App)) OR (Portable Electronic Applications)) OR (Application, Portable Electronic)) OR (Electronic Application, Portable)) OR (Portable Electronic Application))) OR (((((((((((((((((((((((((((((((((Cell Phone) OR (Phone, Cell)) OR (Phones, Cell)) OR (Cellular Phone)) OR (Cellular Phones)) OR (Phone, Cellular)) OR (Phones, Cellular)) OR (Telephone, Cellular)) OR (Cellular Telephone)) OR (Cellular Telephones)) OR (Telephones, Cellular)) OR (Cell Phones)) OR (Portable Cellular Phone)) OR (Cellular Phone, Portable)) OR (Cellular Phones, Portable)) OR (Portable Cellular Phones)) OR (Transportable Cellular Phone)) OR (Cellular Phone, Transportable)) OR (Cellular Phones, Transportable)) OR (Transportable Cellular Phones)) OR (Mobile Phone)) OR (Mobile Phones)) OR (Phone, Mobile)) OR (Phones, Mobile)) OR (Mobile Telephone)) OR (Mobile Telephones)) OR (Telephone, Mobile)) OR (Telephones, Mobile)) OR (Car Phone)) OR (Car Phones)) OR (Phone, Car)) OR (Phones, Car)) OR (Telephone))) OR (((((((Electronic Mail) OR (Mail, Electronic)) OR (Email)) OR (Emails)) OR (E-Mail)) OR (E Mail)) OR (E-Mails))) OR (((((((((((((((((((Internet-Based Intervention) OR (Internet Based Intervention)) OR (Internet-Based Interventions)) OR (Intervention, Internet-Based)) OR (Interventions, Internet-Based)) OR (Web-based Intervention)) OR (Intervention, Web-based)) OR (Interventions, Web-based)) OR (Web based Intervention)) OR (Web-based Interventions)) OR (Online Intervention)) OR (Intervention, Online)) OR (Interventions, Online)) OR (Online Interventions)) OR (Internet Intervention)) OR (Internet Interventions)) OR (Intervention, Internet)) OR (Interventions, Internet)) OR (Internet))) OR ((((((((((Text Messaging) OR (SMS)) OR (Messaging, Text)) OR (Texting)) OR (Textings)) OR (Short Message Service)) OR (Text Messages)) OR (Message, Text)) OR (Messages, Text)) OR (Text Message))) OR (((((((Distance Counseling) OR (Counseling, Distance)) OR (E-Therapy)) OR (E Therapy)) OR (E-Therapies)) OR (E-Counseling)) OR (E Counseling))) OR (((((((((((((telecommunications system) OR (telecommunication system)) OR (web)) OR (web communication)) OR (wireless)) OR (cloud-based)) OR (web-based)) OR (APP)) OR (short-messages-based interaction)) OR (mobile phone text messaging)) OR (phone call)) OR (internet-based)) OR ((distance) AND (counseling))) ) AND TI ( (((((((((((((((((((((((((((((((((((((((((((((Cardiac Surgical Procedures) OR (Surgery, Cardiac)) OR (Surgery, Heart)) OR (Heart Surgery)) OR (Cardiac Surgery)) OR (Procedure, Cardiac Surgical)) OR (Procedures, Cardiac Surgical)) OR (Surgical Procedure, Cardiac)) OR (Surgical Procedures, Cardiac)) OR (Surgical Procedures, Heart)) OR (Cardiac Surgical Procedure)) OR (Heart Surgical Procedures)) OR (Procedure, Heart Surgical)) OR (Procedures, Heart Surgical)) OR (Surgical Procedure, Heart)) OR (Heart Surgical Procedure)) OR (Arterial Switch Operation)) OR (Cardiac Valve Annuloplasty)) OR (Mitral Valve Annuloplasty)) OR (Cardiomyoplasty)) OR (Heart Arrest, Induced)) OR (Circulatory Arrest, Deep Hypothermia Induced)) OR (Heart Bypass, Right)) OR (Fontan Procedure)) OR (Heart Massage)) OR (Heart Transplantation)) OR (Heart-Lung Transplantation)) OR (Heart Valve Prosthesis Implantation)) OR (Transcatheter Aortic Valve Replacement)) OR (Maze Procedure)) OR (Myocardial Revascularization)) OR (Angioplasty, Balloon, Coronary)) OR (Atherectomy, Coronary)) OR (Coronary Artery Bypass +)) OR (Transmyocardial Laser Revascularization)) OR (Norwood Procedures)) OR (Fontan Procedure)) OR (Pericardial Window Techniques)) OR (Pericardiectomy)) OR (Pericardiocentesis)) OR (cardiac surgery)) OR (heart operation)) OR (cardiosurgery)) OR (Heart surgery)) OR (open-heart surgery)) OR (open heart surgery) ) |
| --- |
