# Supplementary material for: Effects of eHealth Interventions on Quality of Life and Psychological Outcomes in Cardiac Surgery Patients: Systematic Review and Meta-analysis
Source: J Med Internet Res. 2022 Aug 16;24(8):e40090. doi: 10.2196/40090 (PMC9428777; doi:10.2196/40090)
Supplement: Multimedia Appendix 3 [file jmir_v24i8e40090_app3.docx]

Multimedia Appendix 3

(Effects of e-health interventions on quality of life and psychological outcomes in cardiac surgery patients: a systematic review and meta-analysis)

##### Table S2: Baseline characteristics of the19 studies selected for the meta-analysis

| Study | Region | Design | Type of surgery | Age (years), mean (SD)/mean (IQR)/mean (range) | Sample (N) | Intervention | Follow-up until |
| --- | --- | --- | --- | --- | --- | --- | --- |
| Chunta [29] | USA | RCT | CABG and/or VR | CG:IG:66.71(range 42-86) | CG:13 IG:15 | telephone | 3months |
| Keeping-Burke et al [30] | Canada | RCT | CABG surgery | not reported | CG:91 IG:91 | Telehealth, home monitoring equipment | 3 weeks |
| Lin et al [31] | Hong Kong,Iran,Sweden, New Zealand, UK | RCT | CABG surgery | CG:75.23±5.82 IG:74.32±5.26 | CG:144 IG:144 | sending reminders via short message services | 18 months |
| Arthur et al [32] | Canada | RCT | CABG surgery | CG:63.8±7.8 IG:61.8±8.4 | CG:123 IG:123 | telephone | 6 months |
| Qu et al [33] | China | RCT | CABG | CG:62.9±8.9 IG:62.4±8.7 | CG:4353 IG:5653 | smartphone | 33 months |
| Rollman et al [34] | USA | RCT | CABG | CG:64±11.2 IG:64±10.8 | CG:152 IG:150 | telephone | 8 months |
| Widmer et al [35] | USA | RCT | PCI | CG:62.5±10.7 IG:63.6±10.9 | CG:34 IG:37 | online and smartphone | 6 months |
| Watanabe et al [36] | Germany, Japan | RCT | Implanted pacemaker | CG:77.2±9.7 IG:75.8±9.7 | CG:638 IG:636 | home monitoring | 24 months |
| Lunde et al [37] | Norway | RCT | PCI CABG Valve surgery Other | CG:58.4±8.2 IG:59.5±9.1 | CG:56 IG:57 | APP | 12 months |
| Martorella et al [38] | Canada | RCT | CABG and/or VR | CG:63.2±9.9 IG:64.6±8.2 | CG:22 IG:30 | Web application | 7days |
| Yu et al [39] | China | RCT | CABG | CG:57.1±9.20 IG:57.4±8.99 | CG:499 IG:501 | Smartphone application | 6 months |
| Barnason et al [40] | USA | RCT | CABG | CG:IG:71.21±4.91 | CG:137 IG:143 | telehealth device | 6 months |
| Bikmoradi et al [41] | Iran, Sweden | RCT | CABG | CG:64.03±7.77 IG:62±7.41 | CG:35 IG:36 | telephone | 5 weeks |
| Danielsen et al [42] | Norway, Belgium, Sweden, South Africa | RCT | aortic valve replacement | CG:67.3±9.8 IG:65.8±11.1 | CG:141 IG:141 | telephone | 12 months |
| Gomis-Pastor et al [43] | Spain | RCT | heart transplants | CG:IG:57±14 | CG:63 IG:71 | mobile application | 12 months |
| Jeon et al [44] | Korea | RCT | cardiac valve replacement | CG:60.07±16.09 IG:59.91±11.60 | CG:17 IG:16 | Application | 5 weeks |
| Lindman et al [45] | USA | RCT | transcatheter aortic valve replacement | CG:76±9 IG:76±7 | CG:25 IG:25 | iPad | 6 months |
| Yadav et al [46] | India；USA | RCT | PCI | CG:54.2±10.2 IG:54.3±11.3 | CG:119 IG:109 | smart phone app | 6weeks |
| Zhu et al [47] | China,GERMANY | RCT | Mechanical heart valve replacement | CG:50.6±9.65 IG:49.59±9.46 | CG:361 IG:360 | a mobile user interface medical network follow-up platform | 12 months |

^a^CG:control group

^b^CABG: coronary artery bypass graft surgery;

^c^IG:intervention group

^d^PCI：Percutaneous coronary intervention ;

^e^RCT: Randomized Controlled Trial;

^f^VR: valve replacement;
