# Supplementary material for: Effects of eHealth Interventions on Quality of Life and Psychological Outcomes in Cardiac Surgery Patients: Systematic Review and Meta-analysis
Source: J Med Internet Res. 2022 Aug 16;24(8):e40090. doi: 10.2196/40090 (PMC9428777; doi:10.2196/40090)

Multimedia Appendix 4

(Effects of e-health interventions on quality of life and psychological outcomes in cardiac surgery patients: a systematic review and meta-analysis)

Figure S1 Forest plot of the effect of e-health on other cardiac postoperative outcomes(Continuous data)


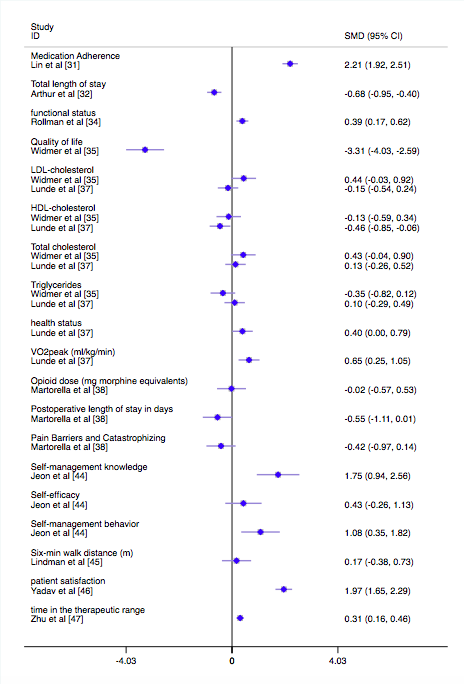

Supplement: Multimedia Appendix 4 [file jmir_v24i8e40090_app4.docx]
