# Supplementary material for: Effects of eHealth Interventions on Quality of Life and Psychological Outcomes in Cardiac Surgery Patients: Systematic Review and Meta-analysis
Source: J Med Internet Res. 2022 Aug 16;24(8):e40090. doi: 10.2196/40090 (PMC9428777; doi:10.2196/40090)
Supplement: Multimedia Appendix 6 [file jmir_v24i8e40090_app6.docx]

Multimedia Appendix 6

(Effects of e-health interventions on quality of life and psychological outcomes in cardiac surgery patients: a systematic review and meta-analysis)

**Table S3 Influence analysis**

|  | **Study omitted** | **Estimate** | **95% Cl** |
| --- | --- | --- | --- |
| **Outcome** |  |  |  |
| **Quality of life-physical SMD** | Arthur et al [32] | 0.19 | 0.05-0.33 |
|  | Rollman et al [34] | 0.14 | -0.004-0.29 |
|  | Bikmoradi et al [41] | 0.14 | 0.13-0.27 |
|  | Lin et al [31] | 0.13 | -0.01-0.28 |
|  | Lunde et al [37] | 0.14 | 0.01-0.27 |
|  | Lindman et al [45] | 0.16 | 0.04-0.29 |
|  |  |  |  |
| **Quality of life-mental** | Arthur et al [32] | 0.19 | 0.03-0.34 |
| **SMD** | Rollman et al [34] | 0.02 | -0.15-0.18 |
|  | Lin et al [31] | 0.08 | -0.09-0.24 |
|  | Lindman et al [45] | 0.11 | -0.03-0.25 |
|  |  |  |  |
| **Depression** | Rollman et al [34] | -0.65 | -1.02- -0.28 |
| **SMD** | Widmer et al [35] | -0.4 | -0.61- -0.18 |
|  | Lindman et al [45] | -0.52 | -0.72- -0.31 |
|  |  |  |  |
