# Supplementary material for: Effects of eHealth Interventions on Quality of Life and Psychological Outcomes in Cardiac Surgery Patients: Systematic Review and Meta-analysis
Source: J Med Internet Res. 2022 Aug 16;24(8):e40090. doi: 10.2196/40090 (PMC9428777; doi:10.2196/40090)
Supplement: Multimedia Appendix 7 [file jmir_v24i8e40090_app7.docx]

Multimedia Appendix 7

(Effects of e-health interventions on quality of life and psychological outcomes in cardiac surgery patients: a systematic review and meta-analysis)

**Tables S4 The overall quality of the evidence for each outcome**

| Certainty assessment | | | | | | | Certainty |
| --- | --- | --- | --- | --- | --- | --- | --- |
| Number of study | Study design | Bias risk | Inconsistency | Indirect | Precision | Other considerations |  |
| Quality of life-physical | | | | | | | |
| 6 | RCT | Serious a | Not serious | Not serious | Not serious | None | ⨁⨁⨁◯ |
|  |  |  |  |  |  |  | Moderate |
|  | | | | | | | |
| Quality of life-mental | | | | | | | |
| 4 | RCT | Serious a | Not serious | Not serious | Not serious | None | ⨁⨁⨁◯ |
|  |  |  |  |  |  |  | Moderate |
|  | | | | | | | |
| Depression | | | | | | | |
| 3 | RCT | Serious a | Serious b | Not serious | Not serious | None | ⨁⨁◯◯ |
|  |  |  |  |  |  |  | 低 |

^a^Downgraded one level due to risk of bias.

^b^Downgraded one level due to inconsistency.
